# A novel approach for estimating the flowering rate of litchi based on deep learning and UAV images

**DOI:** 10.3389/fpls.2022.966639

**Published:** 2022-08-25

**Authors:** Peiyi Lin, Denghui Li, Yuhang Jia, Yingyi Chen, Guangwen Huang, Hamza Elkhouchlaa, Zhongwei Yao, Zhengqi Zhou, Haobo Zhou, Jun Li, Huazhong Lu

**Affiliations:** ^1^College of Engineering, South China Agricultural University, Guangzhou, China; ^2^Guangdong Laboratory for Lingnan Modern Agriculture, Guangzhou, China; ^3^Guangdong Academy of Agricultural Sciences, Guangzhou, China

**Keywords:** litchi flowering rate, convolutional neural network, object detection, UAV images, image analysis

## Abstract

Litchi flowering management is an important link in litchi orchard management. Statistical litchi flowering rate data can provide an important reference for regulating the number of litchi flowers and directly determining the quality and yield of litchi fruit. At present, the statistical work regarding litchi flowering rates requires considerable labour costs. Therefore, this study aims at the statistical litchi flowering rate task, and a combination of unmanned aerial vehicle (UAV) images and computer vision technology is proposed to count the numbers of litchi flower clusters and flushes in a complex natural environment to improve the efficiency of litchi flowering rate estimation. First, RGB images of litchi canopies at the flowering stage are collected by a UAV. After performing image preprocessing, a dataset is established, and two types of objects in the images, namely, flower clusters and flushes, are manually labelled. Second, by comparing the pretraining and testing results obtained when setting different training parameters for the YOLOv4 model, the optimal parameter combination is determined. The YOLOv4 model trained with the optimal combination of parameters tests best on the test set, at which time the mean average precision (mAP) is 87.87%. The detection time required for a single image is 0.043 s. Finally, aiming at the two kinds of targets (flower clusters and flushes) on 8 litchi trees in a real orchard, a model for estimating the numbers of flower clusters and flushes on a single litchi tree is constructed by matching the identified number of targets with the actual number of targets *via* equation fitting. Then, the data obtained from the manual counting process and the estimation model for the other five litchi trees in the real orchard are statistically analysed. The average error rate for the number of flower clusters is 4.20%, the average error rate for the number of flushes is 2.85%, and the average error for the flowering rate is 1.135%. The experimental results show that the proposed method is effective for estimating the litchi flowering rate and can provide guidance regarding the management of the flowering periods of litchi orchards.

## Introduction

Litchi fruit is full of bright, tender and juicy flesh and is one of the four famous types of fruits of south China. In the growth and production of litchi trees, fruit yield is affected by various factors, such as the climate, fertilizer and irrigation (Yang et al., 2015; Zhu, 2020). Many studies have shown that together with the external factors above, the number of litchi flowers, an internal factor, directly impacts litchi fruit yields as well as fruit colour and weight; too many or too few flowers is not conducive to the growth of litchi trees (Lin J. et al., 2022). Therefore, regulating the number of litchi flowers plays a key role in the management of litchi florescence. Statistical litchi flowering rate data can provide an important reference for litchi flower regulation, and they also directly determine the quality and yield of litchi fruit, which have important economic value (Li et al., 2012; Qi et al., 2019).

**FIGURE 1:**
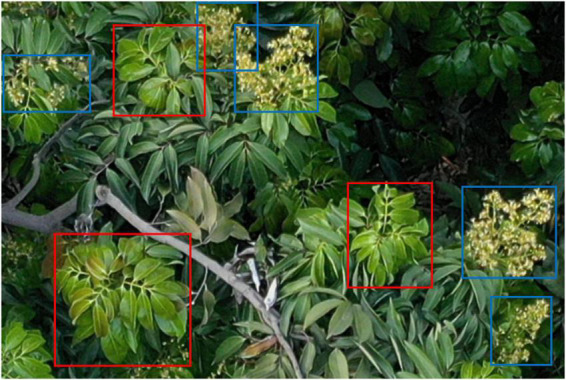
Flower clusters and flushes during the litchi flowering stage.

At present, the statistical work regarding the litchi flowering rate is still based on manual counting, which requires considerable manpower and time (Ren et al., 2021). To save time, farmers use past experience to estimate the litchi flowering rate, which often leads to ineffective litchi flowering management. Therefore, accurate litchi flowering rate statistics are important and can not only provide guidance for the effective management of the flowering period but also reserve nutrition for the tree as needed to facilitate later flowering and fruit setting. The litchi flowering rate is determined by both litchi flower clusters and flushes, as shown in Figure 1, where the blue boxes are the flower clusters to be counted and the red boxes are the flushes to be counted.

In recent years, with the rapid development of artificial intelligence, technologies such as remote sensing have provided great potential for precision agriculture and intelligent agriculture. The development of UAV-based remote sensing systems has taken remote sensing to the next level. Compared to traditional remote sensing techniques, UAVs flying at low altitudes have resulted in ultrahigh spatial resolution images, which has greatly improved the performance of monitoring systems. Furthermore, UAV-based monitoring systems have high temporal resolution, which enhances the flexibility of the image acquisition process. Because of their high spatial resolution, good mobility and flexibility, unmanned aerial vehicles (UAVs) are widely used in many agricultural fields, including plant protection, crop monitoring, and crop yield evaluation (Mukherjee et al., 2019; Tsouros et al., 2019).

In addition, deep learning, as a new research direction in machine learning, has also been developed. Target detection based on deep learning has become a popular research direction in computer vision, and many new target detection algorithms have been proposed by various researchers (Li J. et al., 2021). Compared with traditional target detection algorithms, deep learning-based methods have the advantages of higher speed, higher precision and stronger robustness in complex conditions (Tan et al., 2021; Ren et al., 2022). At present, the commonly used target detection algorithms based on deep learning are mainly divided into one-stage target detection algorithms and two-stage target detection algorithms. The main difference between them is whether they need to generate a regional proposal in advance.

The one-stage target detection algorithms, also known as end-to-end learning-based target detection algorithms, have no steps for generating candidate regions and directly obtain the final detection results; representative one-stage target detection algorithms include YOLO (Redmon et al., 2016; Redmon and Farhadi, 2016, 2018; Bochkovskiy et al., 2020) and single-shot detector (SSD) (Liu et al., 2016). Two-stage target detection algorithms are also known as target detection algorithms based on region nomination. This kind of algorithm first generates candidate regions that may contain objects and then further classifies and calibrates the candidate regions to obtain the final detection results. Representative two-stage target detection algorithms are the region-based convolutional neural network (R-CNN) (Girshick et al., 2013), Fast R-CNN (Girshick, 2015), Faster R-CNN (Ren et al., 2017) and so on.

The advantage of one-stage target detection algorithms is that their detection speeds are high, but the disadvantage is that they have difficulty extracting feature information from small targets, which leads to poor detection effects for local targets. The advantage of two-stage target detection algorithms is that their detection precision is relatively high, but the disadvantage is that their candidate region generation methods and relatively complex network structures lead to low detection speeds, preventing them from achieving real-time detection (Bouguettaya et al., 2022; Ghasemi et al., 2022).

However, equipment combining unmanned aerial systems (UASs) and computer vision technology has gradually become a new type of equipment in modern agricultural engineering.

Some researchers have used UAVs to estimate the yields of crops with low growth heights, such as rice, potato, and wheat (Al-Gaadi et al., 2016; Hassan et al., 2019; Reza et al., 2019; Duan et al., 2021). Li et al. (2017) used UAVs to take rice images from the heading stage to the maturity stage, and the images were used for cluster analysis and image segmentation according to the colour characteristics of rice to obtain the number of rice panicles; this value was input into a yield estimation formula to estimate the rice yield. Li et al. (2020) used a UAV to acquire RGB and hyperspectral imaging data from a potato crop canopy at two growth stages, realizing aboveground biomass and potato crop yield prediction. Zhou Y. et al. (2021) used UAVs to obtain RGB images of wheat booting and flowering periods. Through image processing, the colour and texture feature indices of the wheat images were obtained by analysing their correlations with wheat yield, establishing an effective yield estimation model (Zhou Y. et al., 2021).

To accurately measure the yields of fruit trees, in recent years, some researchers have used UAVs to take fruit images and have combined them with deep learning methods to detect the number of fruits (Barbosa et al., 2021; Zhou X. et al., 2021). Apolo-Apolo et al. (2020) used images captured by a UAV to monitor a total of 20 commercial citrus trees and then developed an automated image processing methodology to detect, count and estimate the sizes of citrus fruits on individual trees using deep learning techniques. Xiong et al. (2020) used UAVs to obtain mango images. Using the You Only Look Once version 2 (YOLOv2) model to conduct training and testing on the mango dataset, the detection precision reached 96.1%, so this approach could estimate the number of mango fruits. Li D. et al. (2021) proposed a scheme based on UAV images and the YOLOv4 model to detect and locate suitable picking points on the fruiting branches of longan. The above studies show that methods based on UAVs and deep learning can solve related problems in agriculture.

In view of the advantages of UAV applications in agriculture and the existing problems regarding litchi flowering rate estimation, this study proposes a scheme based on UAV images and computer vision detection technology. The scheme helps to accurately count the numbers of litchi flower clusters and flushes in natural environments and thus enables the quick calculation of the litchi flowering rate. The scheme can also help to quickly and accurately count the flowering rates of other fruit trees. The main innovations of this study are as follows: (1) the fast statistical litchi flowering rate data acquisition method proposed in this study can provide an important reference for regulating the number of litchi flowers; (2) the effects of different training parameters on the performance of YOLOv4 for detecting flower clusters and flushes are compared; (3) it is determined that the use of a fitting equation to modify the statistical results of YOLOv4 is very effective; and (4) an evaluation of flowering rate estimation schemes is conducted through trials in real orchards. In summary, compared with the results of manual statistics, the average error rates of litchi flower clusters and flushes calculated by the target detection and number estimation models are 4.20 and 2.85%, respectively; the average error between the predicted flowering rate and the actual flowering rate is 1.135%. These results can guide litchi management during the flowering period while providing a reference for the fruit setting and fruit production stages of litchi.

## Materials and methods

### Materials, image acquisition, image preprocessing, image annotation and dataset construction

#### Material samples

**FIGURE 2:**
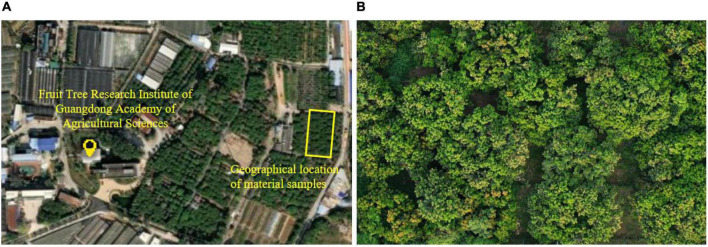
Geographical location of the material samples and the top view of the litchi orchard during the flowering period. **(A)** Geographical location of material samples. **(B)** Top view the of litchi orchard during the flowering period.

The experimental litchi orchard is located within the Institute of Fruit Tree Research, Guangdong Academy of Agricultural Sciences, on Wushan Road, Tianhe, Guangzhou. The average annual temperature at this site is 24–25^°^C. The litchi variety sampled is Guiwei litchi, and its flowering period is generally from March to April. The experimental area is approximately 0.12 hm^2^. The area contains 36 trees with an average age of 26 years. The geographic location information of the sample is shown in Figure 2A, and the top view of the litchi orchard where the samples are located during the flowering period is shown in Figure 2B.

#### Image acquisition equipment

In this study, a DJI Mavic 2 Pro UAV is used for image data acquisition. This UAV has an omnidirectional sensing system, its maximum flight time is 31 min, and the maximum remote control signal distance is 10 km. The sensor is a Hasselblad L1D-20c camera with a focal length of 28 mm, and the aperture value is f/2.8–f/11 (Elkhrachy, 2021).

#### Image acquisition

**FIGURE 3:**
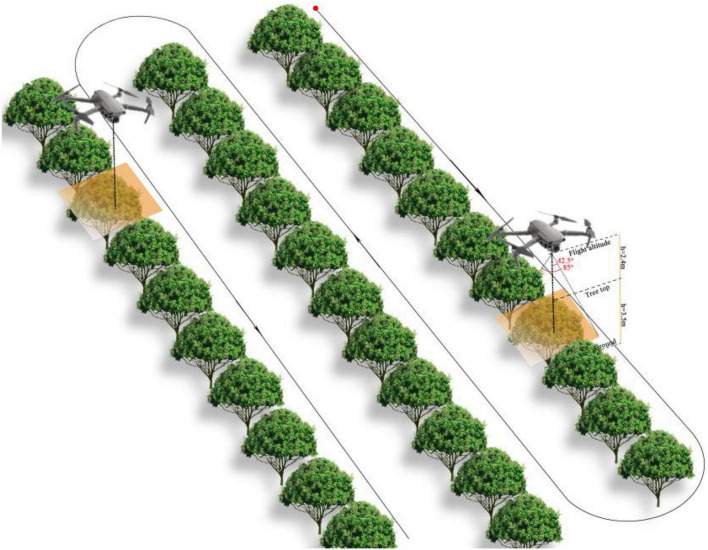
Image acquisition method.

**FIGURE 4:**
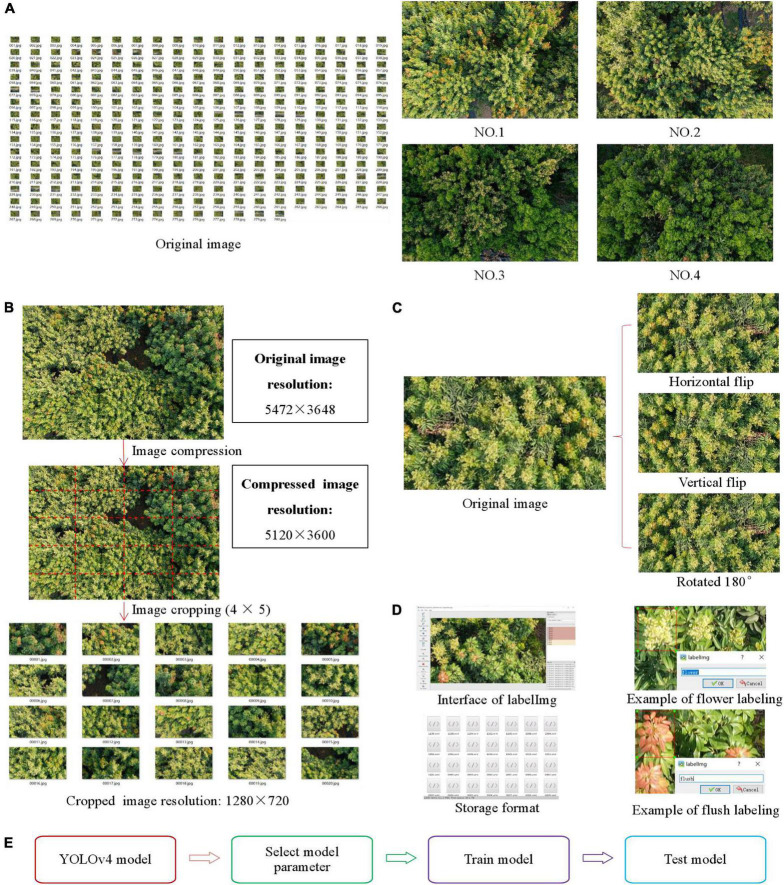
Materials, image acquisition, image preprocessing, image annotation, and model training. **(A)** Image data collected by UAV. **(B)** Image cropping process. **(C)** Data enhancement process. **(D)** Data annotation process. **(E)** Whole training process.

The image data are acquired during two time periods, 8:00–10:00 a.m. and 15:00–17:00 p.m., on clear days from March 15 to April 15, 2021. The image acquisition method is shown in Figure 3. During the image acquisition process, the UgCS software of the UAV ground station is used to plan the flight mission and then it transmits the mission to the UAV, receives the image transmission data returned by the UAV, and completes the image acquisition procedure for the selected litchi tree area. When planning the flight mission, the imaging angle is −90° [that is, the camera lens is perpendicular to the ground (top view)], the flight height is 5.9 m, the flight speed is 1 m/s, the horizontal overlap rate is 70%, and the longitudinal overlap rate is 80%. The UAV acquires 280 images during the litchi flowering period with a resolution of 5472 × 3648, the image format is JPG, and the collected image data are shown in Figure 4A.

#### Image preprocessing

The image preprocessing phase mainly includes two aspects: image cropping and data enhancement. To meet the computational requirements of the model and reduce the computational volume and the computing time of the training model, the images with a resolution of 5472 × 3648 are compressed to 5120 × 3600, and then image cropping is carried out. One image is cropped into 4 × 5 = 20 images, and the resolution of each cropped image is 1280 × 720. The image cropping process is shown in Figure 4B. To ensure the effectiveness of the training model and obtain enough training samples, first, the images that are not clear, such as those that are overexposed and blurred, are screened, and finally, 300 images are selected as the original images of the dataset. Then, the original images are enhanced with three types of data enhancement: horizontal flipping, vertical flipping and 180° rotation. This process increases the generalization ability of the network and reduces the probability of overfitting. The dataset contains 1,200 images after data enhancement. The process of data enhancement is shown in Figure 4C.

#### Image annotation and dataset construction

As this paper uses a supervised deep learning model for target detection, before target detection, the positive and negative samples need to be labelled, that is, the regions of interest (ROIs) in the images need to be manually annotated. In this study, LabelImg software is used to label the samples in the images, and the samples are labelled with two items, flowers (flower clusters) and flushes (flushes). The labelling boxes are rectangles surrounding the ROIs. The image annotation information of the flower clusters and flushes is saved in VOC format, and the corresponding XML files are generated after the labelling procedure (Li et al., 2022; Lin Y. et al., 2022). The XML file contains the image storage information, image name, annotation name and coordinate information of each labelled rectangle box. The data annotation process is shown in Figure 4D.

In Table 1, the numbers of images and pieces of sample information contained in the dataset are counted. The 1,200 images in the dataset are divided into a training set, validation set, and test set at a ratio of 3:1:1. The total number of samples is 47,192, including 38,362 litchi flower cluster samples and 8,830 flush samples.

**TABLE 1:** Numbers of images and pieces of sample information contained in the dataset.

Dataset	Images	Flower_cluster bounding boxes	Flush bounding boxes
Training dataset	720	22908	3579
Validation dataset	240	7863	1841
Testing dataset	240	7591	1638
Complete dataset	1200	38362	8830

### Calculation method for the litchi flowering rate

The flowering rate of a single litchi tree is determined by the numbers of flower clusters and flushes it possesses and is calculated as follows:


(1) Flowering⁢rate⁢of⁢litchi

$$\;=\frac{\mathrm{The}\,\mathrm{number}\,\mathrm{of}\,\mathrm{flower}\,\mathrm{clusters}\,\mathrm{per}\,\mathrm{litchi}}{\begin{array}{c} \text{The number of flower clusters}\\ \text{and the number of flushes per litchi} \end{array}}\times 100\%$$

In the formula, the number of flower clusters per litchi is the total number of flower clusters on a single litchi tree; the number of flushes of a single litchi tree is the total number of single litchi without flower clusters. Flower clusters are branches that have the ability to head and have more than one flower bud; flushes are branches that do not have the ability to head.

### Target detection model and its training process

To detect litchi flower clusters and flushes accurately and in real time, the YOLOv4 model, a one-stage target detection algorithm based on deep learning, is used as the detection algorithm in this paper. First, the ROIs are labelled for 1,200 images in the dataset; then, the labelled images are used for pretraining, and the optimal model parameters of the YOLOv4 model are selected by comparing the obtained precision values; finally, the YOLOv4 model is trained and tested with the selected parameters. The whole training process is shown in Figure 4E.

**FIGURE 5:**
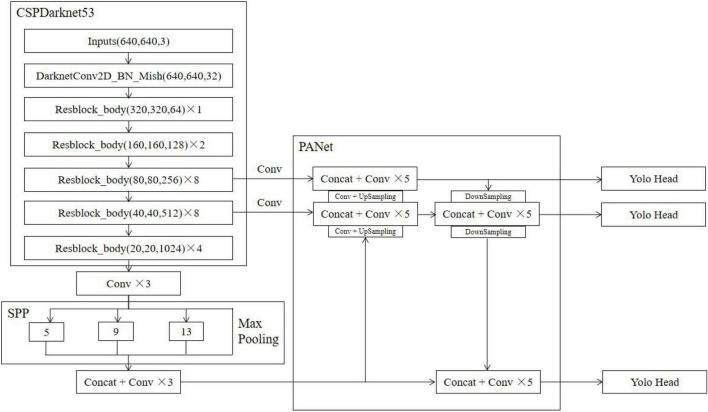
Network model structure diagram of YOLOv4.

The YOLOv4 model consists of four parts: the input, backbone, neck, and head. In this paper, the network structure of the YOLOv4 model is drawn with a 640 × 640 image size as the input size, as shown in Figure 5. Its backbone network is CSPDarknet53, SPP is used as an additional module for the neck, PANet is used as a feature fusion module for the neck, and the head follows the head of YOLOv3. Cross-stage partial (CSP) networks can enhance the learning ability of CNNs, which can maintain precision, reduce computational bottlenecks, and lower memory costs while achieving lightweight computing. CSPDarknet53 adds CSP to each residual block of Darknet53, which increases the depth of the convolution kernel and improves the feature extraction ability of the model for Flower_cluster and Flush. The main role of the SPP network is to increase the perceptual field of the network. PANet adds the DownSample operation after UpSample to improve the effect when feature stitching. In conclusion, the network structure of YOLOv4 further improves the ability to detect Flower_cluster and Flush in orchard scenes.

## Experiments and results

### Model performance evaluation indices

**FIGURE 6:**
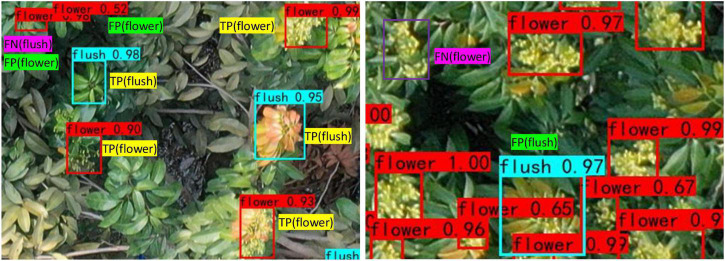
TP, FP, and FN samples of flower clusters and flushes.

At present, many performance evaluation indices are available for target detection based on deep learning. Because this study focuses on whether litchi flower clusters and flushes can be accurately identified, the numbers of correct and incorrect recognition results can be used to evaluate the performance of the model. The precision (P), recall (R), F1-score (F1), average precision (AP), mean AP (mAP), and speed are used in this study. Among them, precision is a statistic from the perspective of prediction results, and it refers to how many of the predicted positive samples are actually positive samples; recall is a statistic from the perspective of the real dataset, and it refers to how many positive samples the model identified out of the total positive samples. F1 is the harmonic average of precision and recall; in this paper, the score threshold is 0.5. The precision, recall and F1 are calculated as follows:


(2) $$P=\frac{T\,P}{T\,P+F\,P}$$

(3) $$R=\frac{T\,P}{T\,P+F\,N}$$

(4) $$F\,1=\frac{2^{*}\,P^{*}\,R}{P+R}$$

where TP means that positive samples are correctly identified as positive samples, FP means that negative samples are wrongly identified as positive samples, and FN means that positive samples are wrongly identified as negative samples. The TP, FP, and FN samples of flower clusters and flushes are shown in Figure 6.

AP is the area enclosed by the PR curve and the coordinates of a single category. This index can comprehensively weigh the precision and recall of the target, and it is a more comprehensive index of the single-category recognition effect of the model. mAP is the average of the AP values obtained for all categories, which reflects the overall detection precision of the model and is the most important performance evaluation index for target detection algorithms.

### Optimization of the model parameters

In the same model training process, parameters such as the size of the input image, the batch size and the number of iterations have great impacts on the detection performance of the utilized model. To obtain the best model performance, the model is pretrained and tested to select the best model parameters. The hardware platform configuration used for the model training and testing experiments includes an Intel (R) Core (TM) i9-10980XE CPU @ 3.00 GHz processor, a 48-GB NVIDIA RTX A6000 graphics card, 32 GB of memory, a 1-TB solid-state drive and a 16-TB hard disk drive. The computer system runs on Ubuntu 18.04, the programming language is Python, and the deep learning framework is PyTorch.

#### Input image size

The input image size has a great impact on the performance of the model. Increasing the input image size is conducive to improving the precision of the model because a reduction in the resolution of the feature map easily leads to a lack of semantic information for small targets; thus, increasing the input resolution will improve the semantic information of the small targets, which is conducive to improving the detection precision for these small targets. However, when the input image size is increased to a certain extent, the detection precision is reduced if the size continues to increase. Because the network structure does not change, the receptive field of the network is also certain. Therefore, if the input image size is increased, the proportion of the receptive field in the image decreases, leading to the local information extracted by the network being unable to effectively predict the targets at all scales and thus causing the detection precision to decrease.

During the pretraining process, the other model parameters are maintained: during the freezing stage, the learning rate is 0.001, the number of epochs is 500, and the number of iterations is 90,000; in the unfreezing stage, the learning rate is 0.0001, the number of epochs is 500, and the number of iterations is 180,000. After changing only the input image size of the YOLOv4 model, information regarding the precision and detection speed of the model performed on the test dataset is shown in Table 2.

**TABLE 2:** mAPs and speeds obtained with different input image sizes.

Resolution of each input image (pixels)	mAP (%)	Speed of detection per image (s)
320 × 320	84.32	0.036
480 × 480	87.09	0.039
640 × 640	87.87	0.043
800 × 800	87.78	0.052
960 × 960	87.61	0.069
1280 × 720	85.61	0.073

As seen from Table 2, when the input image size increases from 320 × 320 to 480 × 480, mAP increases by 2.77%, and when the input image size increases from 480 × 480 to 640 × 640, mAP increases by 0.78%. In these two stages, with the increase in the input image size, the semantic information regarding flower clusters and flushes is constantly enriched, and the detection precision of the model improves. When the input image size increases from 640 × 640 to 800 × 800 and from 800 × 800 to 960 × 960, mAP decreases by 0.09 and 0.17%, respectively. In particular, the original images are used for training and testing, and the mAP values are not the best because the receptive field of the network is limited; beyond a certain extent, the network is unable to predict flower clusters and flush targets at all scales. Overall, when the input image size is 640 × 640 pixels, the detection precision of the model is optimal. Although the detection speed is not the best, it can meet the requirements of real-time operation. A comprehensive measurement of 640 × 640 pixels is chosen as the input image size of the YOLOv4 model in this study.

#### Batch size

The batch size has a significant impact on the convergence speed of model training and random gradient noise. Increasing the batch size within a reasonable range can improve the utilization of memory, require fewer iterations to run an epoch, conduct processing faster than a smaller batch size, and cause fewer training shocks. However, blindly increasing the batch size can lead to insufficient memory capacity. In addition, due to the reduced number of iterations required to run an epoch, the precision of the model is lower than that obtained with a small batch size. Masters and Luschi (2018) tested the performance of models with different batch sizes, and increasing the batch size led to a decline in test performance; the use of a small batch yielded the best training stability and generalization performance; when the batch size was 2 or 4, the performance was optimal (Masters and Luschi, 2018).

The training process of the YOLOv4 model is divided into a freezing stage and an unfreezing stage. During the freezing stage, the backbone of the model is frozen, and the feature extraction network does not change and occupies less video memory. In the unfreezing stage, the backbone of the model is not frozen, the feature extraction network and all parameters of the network change, and the occupied memory is large. Normally, the batch size in the unfreezing stage is larger than that in the freezing stage. At the same time, to meet the memory and computing requirements of the GPU, the batch size can only be set to a power of 2.

This study compares the model precision values obtained with different batch sizes, and the results are shown in Table 3. As seen from Table 3, when the batch size for the unfreezing state of the model is fixed to 2, mAP increases by 0.61% and decreases by 0.06% from 2 to 4 and from 4 to 8 for the freezing state, respectively. When the freezing and unfreezing states of the model are increased from (2, 2) to (4, 2), mAP increases by 0.51%, and when they are increased from (4, 2) to (8, 4) and from (8, 4) to (16, 8), mAP decreases by 1.84 and 1.64%, respectively. This is because increasing the batch size within a reasonable range results in fewer iterations and is beneficial to the stability of training; however, if the batch size is too large, the memory capacity of the computer is insufficient, and all the characteristics of the flower clusters and flushes in each iteration cannot be detected. In summary, when the batch size is 4 in the freezing and unfreezing stages and the batch size is 2 in the unfreezing state, the mAP of the model is 87.87%.

**TABLE 3:** Information regarding the mAPs obtained with different batch sizes.

Batch_size		mAP (%)
Freezing	Unfreezing	
2	2	87.26
4	2	87.87
8	2	87.81
4	4	86.15
8	4	86.03
16	8	84.39

#### Training epochs

**FIGURE 7:**
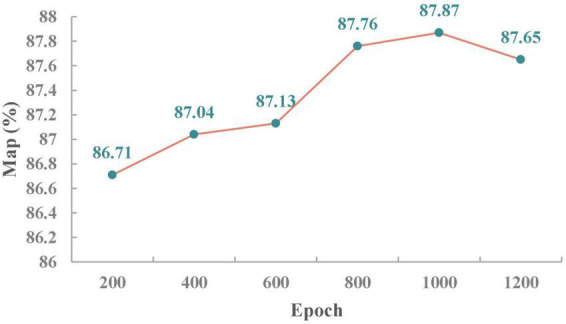
Line chart of the mAP of detection with different numbers of epochs.

An epoch represents the process of training all training samples once. It is not sufficient to transfer the complete dataset once in the neural network; the complete dataset in the same neural network must be transferred many times. However, with the increase in the number of epochs, the times required for updating the weights in the neural network also increase, and the curve changes from underfitting to overfitting. Thus, an appropriate number of epochs is very important for maximizing the precision of the model. As shown in Figure 7, the mAP of the model increases as the number of epochs increases while keeping the other parameters constant because at this time, the network model is gradually optimized and enters the best fitness state. However, when the number of epochs is 1,200, mAP decreases by 0.25% compared with that obtained with 1,000 epochs because the network model enters the overfitting state, which leads to a reduction in network model precision. When the number of epochs is 1,000, the mAP of the model is optimal.

Through the pretraining process and the selection of the YOLOv4 model parameters, when the input image size is 640 × 640, in the freezing state, the batch size is 4, and the learning rate is 0.001; in the unfreezing stage, the batch size is 2, and the learning rate is 0.0001. The number of epochs is 1,000, and mAP is 87.87%. The YOLOv4 model has the best detection precision for flower clusters and flushes on the test dataset. Utilizing the abovementioned experimental hardware platform for testing, the average detection speed is 0.043 s per image, so the proposed approach is able to quickly count the numbers of litchi flower clusters and flushes, thus improving the efficiency of estimating the litchi flowering rate.

### Comparison of model feature extraction capabilities

To further evaluate the feature extraction ability of the YOLOv4 model after preferential parameter selection, the Faster R-CNN, YOLOv4-tiny, CenterNet, and SSD models are trained and tested on the same dataset, and the model performance evaluation indices are analysed and compared with those of the YOLOv4 model. As shown in Table 4, compared with those of other models, the higher R and P values of YOLOv4 show that YOLOv4 not only has fewer missed detections but also fewer false detections, which is a good indication that YOLOv4 has good feature extraction ability. At this time, the AP values of both flower clusters and flushes are higher, and the AP value of flower clusters can reach more than 90%; the overall mAP is also higher. However, Faster-RCNN has the highest R and the lowest P, which indicates that the Faster-RCNN model missed the least detections and misidentified the most detections; CenterNet has the lowest R and the highest P, which indicates that CenterNet missed the most detections and misidentified the least detections. Compared with other models, the YOLO series model has a higher detection speed, and the SSD is also faster than Faster-RCNN, indicating that the one-stage target detection algorithm is more advantageous than the two-stage target detection algorithm. The YOLOv4-tiny model detects each image 0.007 s faster than the YOLOv4 model, which also proves that YOLOv4 still has room for speed improvement. The above results show that after preferential parameter selection for the YOLOv4 model, which is relatively good, this model can be applied to litchi flower rate estimation in real orchards.

**TABLE 4:** Evaluation index results of the test set with different models.

Model	R		P		F1		AP (%)		mAP	Speed of detection per image (s)
	Flower_cluster	Flush	Flower_cluster	Flush	Flower_cluster	Flush	Flower_cluster	Flush	(%)	
Faster R-CNN	85.5	82.48	74.16	71.67	0.79	0.77	82.56	79.27	80.91	0.093
YOLOv4-tiny	70.92	63.17	86.37	83.31	0.78	0.72	85.59	79.57	82.58	0.036
CenterNet	75.95	74.91	90.96	84.68	0.83	0.79	87.45	82.26	84.85	0.064
SSD	82.48	80.16	83.98	76.87	0.83	0.78	87.34	83.39	85.37	0.078
YOLOv4 (this paper)	85.39	74.73	86.96	83.27	0.86	0.79	90.96	84.78	87.87	0.043

### Models for estimating the numbers of flower clusters and flushes on a single litchi tree

To obtain an accurate flowering rate for a single litchi tree, the numbers of flower clusters and flushes detected by the model need to be corrected. First, eight litchi trees are randomly selected from the standard orchard, and the actual numbers of flower clusters and flushes on each litchi tree are calculated manually. Then, the images of each litchi tree are captured from the top of the UAV, and the model trained in the previous section is used to detect the numbers of flower clusters and flushes on each litchi tree. Finally, the numbers produced by manual statistics and the numbers detected by the model are fitted with separate equations to construct a model for estimating the numbers of flower clusters and flushes on a single litchi tree.

When using YOLOv4 to detect the number of flower clusters and flushes on each litchi tree, as the images are obtained from a top view by the UAV, the edge parts of litchi trees appear to shade each other in the images. Therefore, combined with the manual statistics of the specific area of a single litchi tree. Photoshop is used to crop the target litchi images from the original images. To further improve the detection precision of YOLOv4, first, the original images are cropped by 4 × 5, and then the cropped images are detected so that the numbers of flower clusters and flushes on each tree can be obtained more accurately.

**FIGURE 8:**
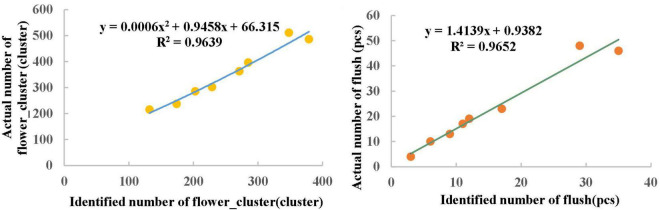
Fitting results for the numbers of flower clusters and flushes on eight litchi trees.

Table 5 provides information regarding the numbers produced by manual counting and YOLOv4 model detection. For the eight litchi trees, the actual range of the number of flower clusters is 215–511, and the actual range of the number of flushes is 4–48. This is because the eight trees are randomly selected and vary in their tree shapes and ages. In addition, the flowering rates of litchi trees are affected by external conditions, such as the available nutrients and light conditions; as a result, the flowering rates of each tree are different. Exponential fitting, linear fitting, logarithmic fitting, binomial fitting and power fitting are used to fit the corresponding flower cluster and flush data in Table 5. According to the comprehensive analysis and comparison results, as shown in Figure 8, the best fitting method for flower clusters is binomial fitting, the fitting equation is *y*0.0006x^2^+0.9458x+66.315, and the coefficient of determination *R*^2^ is 0.9639; the best fitting method for flushes is linear fitting, the fitting equation is *y*1.4139x+0.9382, and the coefficient of determination *R*^2^ is 0.9652. A strong correlation is observed between the two types of samples.

**TABLE 5:** Quantitative information for the manual counts and the YOLOv4 model detection results.

Number	Actual number of flower_clusters (clusters)	Identified number of flower_clusters (clusters)	Actual number of flushes (pcs)	Identified number of flushes (pcs)
1	237	174	10	6
2	215	132	4	3
3	302	229	13	9
4	486	379	48	29
5	285	203	19	12
6	396	285	17	11
7	363	271	46	35
8	511	348	23	17

### Analysis of the test results obtained for a real orchard

#### Test results obtained with different lighting conditions

Different lighting conditions can affect the detection effect of the YOLOv4 model. The image brightness obtained with front lighting is stronger than that obtained with back lighting. In this study, 48 images are collected from the real orchard scene and divided into two situations, front lighting and back lighting, with 24 images in each; this is done to analyse the influence of different lighting conditions on the detection results of the YOLOv4 model.

**FIGURE 9:**
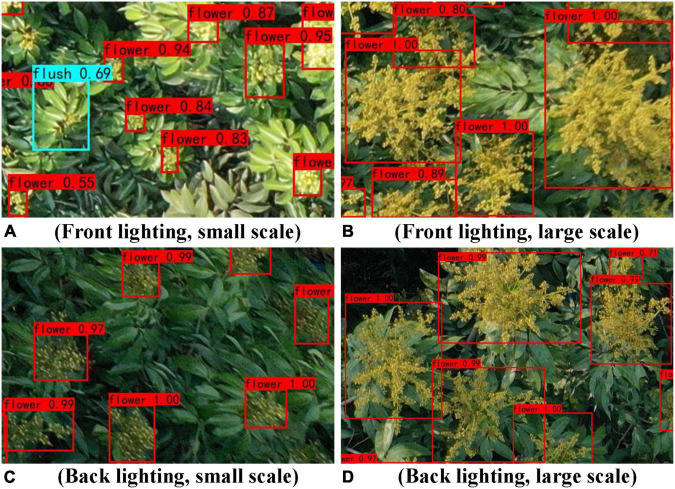
Results of the YOLOv4 model detection results obtained under different lighting conditions.

Table 6 shows the precision information of the YOLOv4 model detection process under different lighting conditions. From the data in Table 6, compared with those under front lighting conditions, the AP of flower_cluster, the AP of flushes, and the mAP of the YOLOv4 model under back lighting conditions are 2.92, 1.57, and 2.24% higher, respectively. From the YOLOv4 model detection results obtained under different lighting conditions (shown in Figure 9), the YOLOv4 model has better detection results for both types of targets in the back lighting scenario because the brightness of front lighting is too strong, which leads to image overexposure. In particular, overexposure of flower clusters diminishes the colour component differences between them and the litchi leaves, resulting in less distinct differences between the edges of the flower clusters and the litchi leaves; the YOLOv4 model has difficulty recognizing the flower clusters in such cases. In contrast, although the image brightness of back lighting is much weaker, the colour components of the flower clusters and litchi flushes are more obvious and easier to distinguish as the images are taken in sunny weather. Therefore, for future sample data collection or research, trials in clear weather should be avoided, and cloudy or overcast days should be chosen whenever possible.

**TABLE 6:** Precision information of the YOLOv4 model detection results obtained under different lighting conditions.

Illumination conditions	Ground_truth		R (%)		P (%)		F1		AP (%)		mAP (%)
	Flower_cluster	Flush	Flower_cluster	Flush	Flower_cluster	Flush	Flower_cluster	Flush	Flower_cluster	Flush	
Front lighting	743	116	86.27	67.24	86.27	85.71	0.86	0.75	91.30	83.83	89.81
Back lighting	526	45	89.54	75.56	89.04	82.93	0.89	0.79	94.22	85.40	87.57

#### Test results obtained with different sparseness levels

The heading sparseness of litchi varies in different areas of the same tree depending on their different nutritional and lighting conditions, especially at the top of the canopy, where the heading ability of litchi tends to be stronger. In this study, 48 images are collected and divided into sparse and dense groups according to the sparseness of the flower clusters and flushes in real orchard scenes, with 24 images in each group. The influence of different sparseness levels on the detection results of the YOLOv4 model is analysed.

**FIGURE 10:**
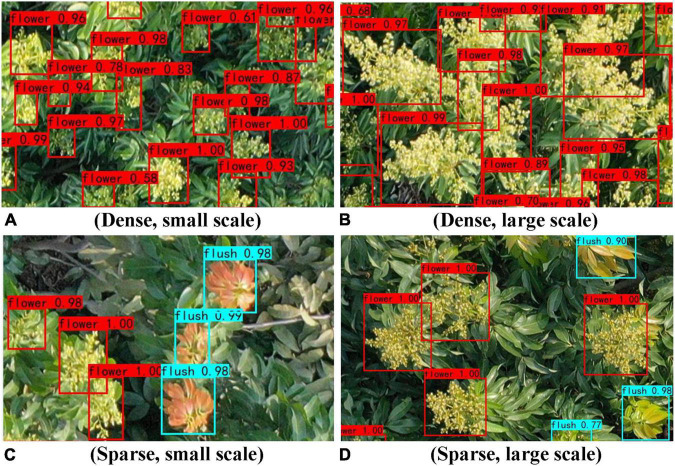
Results of YOLOv4 model detection obtained under different sparseness conditions.

Table 7 shows the precision information of the YOLOv4 model detection results obtained under different density conditions. According to the data in Table 7, compared with that of dense condition, the overall effect of the sparse condition is better, and mAP of model detection for sparse images is 9.68% higher than that for dense images. From the YOLOv4 model detection results obtained under different sparseness conditions (shown in Figure 10), the results for two types of targets are better in sparse scenes. This is mainly due to the occlusion and overlap of dense images, which lead to false model detection, thus reducing the precision of the model. In contrast, for sparse images, most of the samples can be separated clearly without being covered by other samples. In addition, the backgrounds of sparse images are relatively simple and their edge features are good; thus, the model has good performance for sparse images.

**TABLE 7:** Precision information of the YOLOv4 model detection results obtained under different sparseness conditions.

Densities	Ground_truth		R (%)		P (%)		F1		AP (%)		mAP (%)
	Flower_cluster	Flush	Flower_cluster	Flush	Flower_cluster	Flush	Flower_cluster	Flush	Flower_cluster	Flush	
Sparse	358	69	89.11	81.16	90.11	83.58	0.85	0.90	94.03	89.09	91.56
Dense	1274	79	85.09	63.29	85.83	71.43	0.67	0.82	90.52	73.25	81.88

#### Error analysis of the flowering rate

To further validate the estimation model in section “Models for estimating the numbers of flower clusters and flushes on a single litchi tree,” five additional varieties of litchi trees, including Guiwei, Huaizhi, and Nuomici, are randomly selected from the real orchard, and the actual numbers of flower clusters and flushes of each tree are obtained by manual counting. A UAV is used to collect the images, and the YOLOv4 model is used to calculate the numbers of flower clusters and flushes on each tree. According to the numbers of flower clusters and flushes calculated by the YOLOv4 model, the predicted numbers of clusters and flushes are obtained *via* correction by using the fitting equations in section “Models for estimating the numbers of flower clusters and flushes on a single litchi tree.” Then, the flowering rate of each litchi tree is calculated according to the actual number and the predicted number, and the error is analysed. The error in this study is the absolute value of the actual number minus the predicted number, and the error rate is equal to the percentage ratio of this error divided by the actual number.


(5) $$\mathrm{Error}\,\mathrm{rate}=\left|\frac{\mathrm{Actual}\,\mathrm{number}-\mathrm{Predicted}\,\mathrm{number}}{\mathrm{Actual}\,\mathrm{number}}\right|\times 100\%$$

**FIGURE 11:**
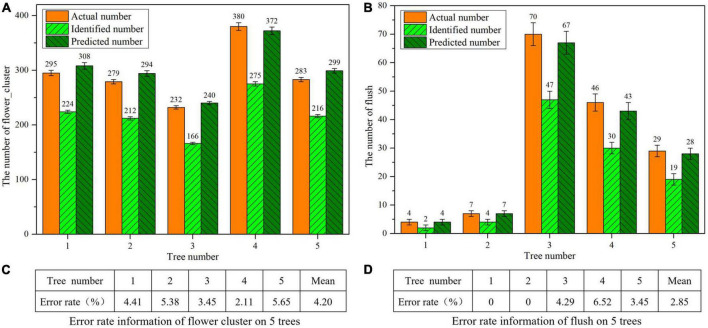
Statistical information of the flower clusters and flushes of 5 litchi trees. **(A)** Statistical information of the flower clusters. **(B)** Statistical information of the flushes. **(C)** Error rates of flower clusters. **(D)** Error rates of flushes.

The actual numbers, identified numbers and predicted numbers of flower clusters and flushes of 5 litchi trees are shown in Figures 11A,B, and their error rates are shown in Figures 11C,D. As seen from the data in Figures 11A,B, the predicted numbers are very close to the actual numbers after the numbers of flower clusters and flushes identified by the YOLOv4 model are fitting by the fitting equations obtained in section “Models for estimating the numbers of flower clusters and flushes on a single litchi tree.” According to the data in Figure 11C, the average error rate of the flower flushes for the five trees is 4.20%. According to the data in Figure 11D, the average error rate of the treetop is 2.85%. From the data in Figures 11C,D, the error rates for both flower clusters and flushes on five litchi trees representing three varieties are less than 7%, which fully illustrates the wide applicability of the YOLOv4 model for estimating the numbers of flower clusters and flushes. The combination of flower clusters and flushes is suitable for estimating the flowering rate of multiple varieties of litchi trees.

According to the statistical results of section “Models for estimating the numbers of flower clusters and flushes on a single litchi tree” and this section, two main reasons are responsible for the error between actual numbers and identified numbers. (1) The mAP of the model on the test dataset is 87.87%; that is, the model itself has error in the identification process. (2) The actual numbers of flower clusters and flushes are obtained by manual counting, which is a multiangle and full-range process, while the UAV collects images from the top, only obtaining flower clusters and flushes from most of the area of a single litchi tree.

Table 8 shows statistics on the real flowering rates of five randomly selected litchi trees in a real orchard and the flowering rates yielded by the proposed method, as well as the error values between them. The error rates between the two types of flowering rates are all below 5%, and the average error is 1.135%. The above results fully prove that the model proposed in this study is feasible. It can not only modify the predicted numbers of flower clusters and flushes by fitting equations but also improve the efficiency of flowering rate estimation in real orchards.

**TABLE 8:** Information on the actual and predicted flowering rates of the five trees and their errors.

Tree number	Actual number of flower_clusters (cluster)	Actual number of flushes (pcs)	Actual flowering rate (%)	Predicted number of flower_clusters (cluster)	Predicted number of flushes (pcs)	Predicted flowering rate (%)	Error of flowering rate (%)
1	295	4	98.662	308	4	98.7	0.038
2	279	7	97.552	294	7	97.62	0.068
3	232	70	76.821	240	67	72.08	4.741
4	380	46	89.202	372	43	88.44	0.762
5	283	29	90.705	299	28	90.64	0.065
Mean	/	/	/	/	/	/	1.135

## Conclusion

In this study, a method based on UAV images and computer vision technology is proposed to estimate the flowering rate of litchi. First, a UAV is used to collect RGB images, preprocess the images, construct a dataset, and manually label the ROIs of the dataset images. Then, the optimal parameters, such as the input image size, batch size and number of epochs, of the YOLOv4 model are optimized based on the model pretraining and testing results. Finally, the YOLOv4 model is trained with the optimized parameter combinations and tested on the test dataset. The results show that the YOLOv4 model can accurately and efficiently detect flower clusters and flushes of litchi with an mAP of 87.87% and an average detection speed of 0.043 s per image. To accurately predict the flowering rate of each litchi tree, a model for estimating the numbers of flower clusters and flushes of litchi is proposed. A fitting equation is established through the actual numbers and predicted numbers of flower clusters and flushes for each litchi tree. The model is verified with average error rates of 4.20% for flower clusters, 2.85% for flushes and 1.135% for the flowering rate. This method can quickly and accurately estimate the flowering rate of litchi, provide guidance for the management of the flowering periods in litchi orchards, and help reduce the labour costs of orchard management.

In this study, UAVs mainly collect images by looking down and cannot obtain omnidirectional images of a single litchi tree or a certain area. Therefore, errors are induced between the collected data and the actual values. In future research, the 3D UAV modelling method will be considered to solve this problem. In model detection, due to the similar colors of new leaves and old leaves, the diversity of litchi flower cluster growth and the presence of complex backgrounds, future consideration should be given to expanding the dataset samples and improving the model to improve its overall detection precision. In addition, the research object of this paper is flower clusters and flushes of Guiwei litchi. In the future, the research object can be extended to different litchi varieties or even other fruit trees. The research method of this paper can also be used for the study of fruit setting rate and yield of fruits.

## Data availability statement

The raw data supporting the conclusions of this article will be made available by the authors, without undue reservation.

## Author contributions

PL and DL conceived and designed the concept and methodology. YJ and HE conducted the experiments. YC and ZY analyzed the data and prepared the figures and tables. ZZ and HZ finished the algorithms and methods. PL wrote the manuscript. HL, JL, GH, and DL supervised the manuscript and made valuable inputs. All authors read and approved the submission of the manuscript.

## References

[B1] Al-Gaadi K. A. Hassaballa A. A. Tola E. Kayad A. G. Madugundu R. Alblewi B. (2016). Prediction of potato crop yield using precision agriculture techniques. *PLoS One* 11:e0162219. 10.1371/journal.pone.0162219 27611577 PMC5017787

[B2] Apolo-Apolo O. E. Martínez-Guanter J. Egea G. Raja P. Pérez-Ruiz M. (2020). Deep learning techniques for estimation of the yield and size of citrus fruits using a UAV. *Eur. J. Agron.* 115:126030. 10.1016/j.eja.2020.126030

[B3] Barbosa B. D. S. Ferraz G. A. E. S. Costa L. Ampatzidis Y. Vijayakumar V. (2021). UAV-based coffee yield prediction utilizing feature selection and deep learning. *Smart Agric. Technol.* 1:100010. 10.1016/j.atech.2021.100010

[B4] Bochkovskiy A. Wang C. Liao H. M. (2020). Yolov4: Optimal speed and accuracy of object detection. *arXiv* [Preprint]. 10.48550/arXiv.2004.10934 35895330

[B5] Bouguettaya A. Zarzour H. Taberkit A. M. Kechida A. (2022). A review on early wildfire detection from unmanned aerial vehicles using deep learning-based computer vision algorithms. *Signal Process.* 190:108309. 10.1016/j.sigpro.2021.108309

[B6] Duan B. Fang S. Gong Y. Peng Y. Wu X. Zhu R. (2021). Remote estimation of grain yield based on UAV data in different rice cultivars under contrasting climatic zone. *Field Crops Res.* 267:108148. 10.1016/j.fcr.2021.108148

[B7] Elkhrachy I. (2021). Accuracy assessment of low-cost unmanned aerial vehicle (UAV) photogrammetry. *Alex. Eng. J.* 60 5579–5590. 10.1016/j.aej.2021.04.011

[B8] Ghasemi Y. Jeong H. Ho Choi S. Park K.-B. Lee J. Y. (2022). Deep learning-based object detection in augmented reality: A systematic review. *Comput. Ind.* 139:103661. 10.1016/j.compind.2022.103661

[B9] Girshick R. (2015). “Fast r-CNN,” in *2015 IEEE International Conference on Computer Vision (ICCV)*, (Santiago: IEEE), 10.1109/ICCV.2015.169

[B10] Girshick R. Donahue J. Darrell T. Malik J. (2013). “Rich feature hierarchies for accurate object detection and semantic segmentation,” in *IEEE Conference On Computer Vision and Pattern Recognition (CVPR)*, (Columbus, OH: IEEE).

[B11] Hassan M. A. Yang M. Rasheed A. Yang G. Reynolds M. (2019). A rapid monitoring of ndvi across the wheat growth cycle for grain yield prediction using a multi-spectral UAV platform. *Plant Sci.* 282 95–103. 10.1016/j.plantsci.2018.10.022 31003615

[B12] Li A. Wang Y. Cao Y. Yu F. Xu T. (2017). Rice yield estimation based on high-definition digital image of UAV. *J. Shenyang Agric. Univ.* 48 629–635. 10.3969/j.issn.1000-1700.2017.05.017

[B13] Li B. Xu X. Zhang L. Han J. Bian C. Li G. (2020). Above-ground biomass estimation and yield prediction in potato by using UAV-based RGB and hyperspectral imaging. *ISPRS J. Photogramm. Remote Sens.* 162 161–172. 10.1016/j.isprsjprs.2020.02.013

[B14] Li D. Sun X. Elkhouchlaa H. Jia Y. Yao Z. (2021). Fast detection and location of longan fruits using UAV images. *Comput. Electron. Agric.* 190:106465. 10.1016/j.compag.2021.106465

[B15] Li G. Suo R. Zhao G. Gao C. Fu L. (2022). Real-time detection of kiwifruit flower and bud simultaneously in orchard using yolov4 for robotic pollination. *Comput. Electron. Agric.* 193:106641. 10.1016/j.compag.2021.106641

[B16] Li J. Li C. Fei S. Ma C. Chen W. Ding F. (2021). Wheat ear recognition based on retinanet and transfer learning. *Sensors* 21:4845. 10.3390/s21144845 34300585 PMC8309814

[B17] Li Z. Yuan P. Qiu Y. Li J. Fan C. (2012). The relationship between winter irrigation and spring flowering of guiwei litchi trees. *Chin. J. Trop. Crops* 33 402–407. 10.3969/j.issn.1000-2561.2012.03.002

[B18] Lin J. Li J. Yang Z. Lu H. Ding Y. Cui H. (2022). Estimating litchi flower number using a multicolumn convolutional neural network based on a density map. *Precis. Agric.* 23 1226–1247. 10.1007/s11119-022-09882-7

[B19] Lin Y. Chen T. Liu S. Cai Y. Shi H. (2022). Quick and accurate monitoring peanut seedlings emergence rate through UAV video and deep learning. *Comput. Electron. Agric.* 197:106938. 10.1016/j.compag.2022.106938

[B20] Liu W. Anguelov D. Erhan D. Szegedy C. Reed S. (2016). “SSD: Single shot multibox detector,” in *Computer Vision – ECCV 2016*, eds Leibe B. Matas J. Sebe N. Welling M. (Cham: Springer), 21–37. 10.1007/978-3-319-46448-0_2

[B21] Masters D. Luschi C. (2018). Revisiting small batch training for deep neural networks. *arXiv* [Preprint]. 10.48550/arXiv.1804.07612 35895330

[B22] Mukherjee A. Misra S. Raghuwanshi N. S. (2019). A survey of unmanned aerial sensing solutions in precision agriculture. *J. Netw. Comput. Appl.* 148:102461. 10.1016/j.jnca.2019.102461

[B23] Qi W. Chen H. Tao L. Fengxian S. (2019). Development status, trend and suggestion of litchi industry in mainland China. *Guangdong Agric. Sci.* 46 132–139. 10.16768/j.issn.1004-874X.2019.10.020

[B24] Redmon J. Divvala S. Girshick R. Farhadi A. (2016). You only look once: Unified, real-time object detection. *Proc. IEEE Conf. Comput. Vis.* 5 779–788.

[B25] Redmon J. Farhadi A. (2016). “Yolo9000: Better, faster, stronger,” in *IEEE Conference on Computer Vision and Pattern Recognition (CVPR)*, (Honolulu, HI: IEEE).

[B26] Redmon J. Farhadi A. (2018). Yolov3: An incremental improvement. *arXiv* [Preprint]. 10.48550/arXiv.1804.02767 35895330

[B27] Ren P. Ma X. Wei B. Wang H. (2021). Effects of foliar rare earth fertilizer on photosynthesis, flowering and fruiting in *Litchi chinensis*. *J. Fruit Sci.* 38 1540–1549. 10.13925/j.cnki.gsxb.20210056

[B28] Ren S. He K. Girshick R. Sun J. (2017). Faster r-CNN: Towards real-time object detection with region proposal networks. *IEEE Trans. Pattern Anal. Mach. Intell.* 39 1137–1149. 10.1109/TPAMI.2016.2577031 27295650

[B29] Ren X. Sun M. Zhang X. Liu L. Zhou H. (2022). An improved mask-rcnn algorithm for UAV tir video stream target detection. *Int. J. Appl. Earth Obs. Geoinf.* 106:102660. 10.1016/j.jag.2021.102660

[B30] Reza M. N. Na I. S. Baek S. W. Lee K. (2019). Rice yield estimation based on k-means clustering with graph-cut segmentation using low-altitude UAV images. *Biosyst. Eng.* 177 109–121. 10.1016/j.biosystemseng.2018.09.014

[B31] Tan L. Lv X. Lian X. Wang G. (2021). Yolov4_drone: UAV image target detection based on an improved yolov4 algorithm. *Comput. Electr. Eng.* 93:107261. 10.1016/j.compeleceng.2021.107261

[B32] Tsouros D. C. Bibi S. Sarigiannidis P. G. (2019). A review on UAV-based applications for precision agriculture. *Information* 10:349. 10.3390/info10110349

[B33] Xiong J. Liu Z. Chen S. Liu B. Zheng Z. Zheng Z. (2020). Visual detection of green mangoes by an unmanned aerial vehicle in orchards based on a deep learning method. *Biosyst. Eng.* 194 261–272. 10.1016/j.biosystemseng.2020.04.006

[B34] Yang B. Li G. Yang S. He Z. Zhou C. Yao L. (2015). Effect of application ratio of potassium over nitrogen on litchi fruit yield, quality, and storability. *Hortscience* 50 916–920. 10.21273/HORTSCI.50.6.916 35581909

[B35] Zhou X. Lee W. S. Ampatzidis Y. Chen Y. Peres N. Fraisse C. (2021). Strawberry maturity classification from UAV and near-ground imaging using deep learning. *Smart Agric. Technol.* 1:100001. 10.1016/j.atech.2021.100001

[B36] Zhou Y. Wang D. Chen C. Li R. Li D. (2021). Prediction of wheat yield based on color index and texture feature index of unmanned aerial vehicle rgb image. *J Yangzhou Univ.* 42 110–116. 10.16872/j.cnki.1671-4652.2021.03.017

[B37] Zhu X. (2020). analysis of suitable climate resources for the growth of high quality litchi in yulin city. *J. Agric. Catastrophol.* 10 126–127. 10.19383/j.cnki.nyzhyj.2020.03.052

